# Report of Case of Puerperal Convulsions

**Published:** 1890-09

**Authors:** W. L. Patten

**Affiliations:** Pensacola, Fla.


### REPORT OF CASE OF PUERPERAL CONVULSIONS.

By W. L. PATTEN, M. D., Pensacola, Fla.

Called to see Mrs. M., aged twenty years; married eleven months; primipara. Found her in puerperal convulsions, which began at 8 p. m., preceding day, with intervals of not more than twenty-five or thirty minutes. It being six miles from the city, they were unable to procure medical aid earlier. I administered chloroform till paroxysm discontinued. Made vaginal examination, found cervix dilated to near the size of a silver dollar. Pelvis very capacious. Size of fœtal head and presentation normal. Complete uterine inertia. Had another convulsion before completing the examination. It was soon checked by inhalation of chloroform as before. Patient had been totally unconscious ever since one hour after onset of first convulsion.

On inquiry the husband informed me that he was not expecting her confinement for at least twenty days hence, and the convulsions came on about two hours after violent exercise and severe fright, with no premonitory symptoms of labor.

I used the ordinary remedies for exciting uterine contraction, but none had the desired effect.

I worked, waited and used those remedies till 7 o'clock p. m., convulsions occurring at regular intervals of about thirty minutes. I found natural delivery impossible and use of forceps inevitable. I dispatched for Dr. F. G. Renshaw, of this city. When he arrived with forceps we immediately delivered her of a dead, but well developed fœtus, full term.

She had a convulsion ten minutes before delivery, but none afterwards.

Removed placenta in few minutes, as uterine contraction was not sufficient to expel it.

Patient was now very much exhausted, pulse very feeble, pupils dilated, face and lips very much cyanosed. In fact complete coma was present.

Instructed the nurses to stimulate her freely with brandy and mustard, and to use turpentine stupes to the umbilical and hypogastric regions, with vaginal injections of hot water every four hours. No antiseptics used.

I left her at 2 A. M. Saw her again at 3 P. M. No apparent change. Continued treatment with addition of

℞. Hydrarg chlor. mitis........................gr. ii.
Sacchari albi....................................xii.
Sodii bicarb............................aa, gr. xii.
M. ft. Pulv. xij.

Sig.—One every two hours.

Saw her at 11 o'clock next day, still comatose; passing urine and fæces involuntarily, with cold clammy perspiration over the body. Made no change in treatment. Saw her again at 6 P. M. No improvement, At 12 o'clock M. next day some improvement, still unconscious. It was now two and a half days after delivery. She gradually improved, and on the fourth day for the first time aroused from her stupor and asked a few questions; but in a semi-rational manner. In a few hours went into a stupor again. She aroused the next day (fifth) with preceding symptoms very much improved, but complained of great cephalalgia. She now began to observe things around her and speak to different members of the family by name. Asked "why the doctor was there and who was sick," and to let her get up and walk about, etc. She remembered nothing that transpired during the five days preceding, knew nothing of the birth of her child, or suspected anything of the kind, till I informed her on the ninth day. She seemed to be very much surprised, but attached very little importance to it and never mentioned it afterwards, showing she had no knowledge whatever of what had transpired. Nothing unpleasant in the case now except increased cephalalgia, which was very severe. Had rested almost none for ten days, except when under the influence of a norcotic or anodyne, Battle's Bromidia having best effects of anything used.

Convalescence very good indeed, no bad symptoms arising. At no time did the temperature go above what we would term normal in the parturient period. Lochia was perfect all the time after third day.

I used tonic treatment and nourishing diet after she regained consciousness. Patient is now in excellent health and mind clear.

